# Immunological aspects and quality of life among patients with lichen sclerosus

**DOI:** 10.1093/skinhd/vzag081

**Published:** 2026-06-15

**Authors:** Witold Chmielnicki, Agata Sidor, Magdalena Grykin, Marta Kasprowicz-Furmańczyk, Agnieszka Owczarczyk-Saczonek

**Affiliations:** Department and Clinic of Dermatology, Sexually Transmitted Diseases and Clinical Immunology, University of Warmia and Mazury, Olsztyn, Poland; Department and Clinic of Dermatology, Sexually Transmitted Diseases and Clinical Immunology, University of Warmia and Mazury, Olsztyn, Poland; Department and Clinic of Dermatology, Sexually Transmitted Diseases and Clinical Immunology, University of Warmia and Mazury, Olsztyn, Poland; Department and Clinic of Dermatology, Sexually Transmitted Diseases and Clinical Immunology, University of Warmia and Mazury, Olsztyn, Poland; Dermatology, Sexually Transmitted Diseases and Clinical Immunology Clinic, The Municipal Polyclinical Hospital, Olsztyn, Poland; Department and Clinic of Dermatology, Sexually Transmitted Diseases and Clinical Immunology, University of Warmia and Mazury, Olsztyn, Poland; Dermatology, Sexually Transmitted Diseases and Clinical Immunology Clinic, The Municipal Polyclinical Hospital, Olsztyn, Poland

## Abstract

Lichen sclerosus (LS) is a chronic, inflammatory dermatosis that predominantly affects the anogenital region. The ethology of LS is currently regarded as multifactorial, encompassing genetic, autoimmune and tissue remodelling factors, as well as oxidative stress or urine occlusion. Clinical manifestations such as white plaques, atrophic skin, erythema, erosions or varying degrees of sclerosis substantially impair patients’ quality of life. It has been indicated that individuals with LS frequently experience feelings of embarrassment, anxiety and social stigma, often resulting from the incorrect association of LS with sexually transmitted diseases. Moreover, the occurrence of sexual dysfunction in this group represents an important clinical concern: patients often refrain from sexual activity due to embarrassment caused by the appearance of the disease, as well as the discomfort associated with it. Recent research indicates that the diminished quality of life observed in patients with LS may arise not only from the direct physical manifestations of the disease, but also from the persistent inflammatory state associated with it. Proinflammatory cytokines, such as interleukin-1, interferon-γ and tumour necrosis factor-α, produced in excessive amounts may adversely influence neurotransmission, thereby contributing to a higher prevalence of psychiatric disorders among patients with LS. The impact appears to involve effects on the metabolism of dopamine, serotonin and noradrenaline. These mechanisms provide a basis for analysing the impact of biologic treatment on patients’ mental health. This association highlights the potential for improving patients’ outcomes, with regard to somatic symptoms and mental health, through the implementation of targeted anti-inflammatory therapeutic strategies.

## Teaser

Based on data from the Lifelines cohort, 3.0% of 28 791 Dutch adults were found to have atopic multimorbidity. Childhood pet ownership, whether cats or dogs, was associated with a lower likelihood of atopic multimorbidity, whereas caesarean section delivery was associated with a higher likelihood. These findings provide insights into potential preventive strategies.

## Graphical Abstract

**Graphical Abstract:**
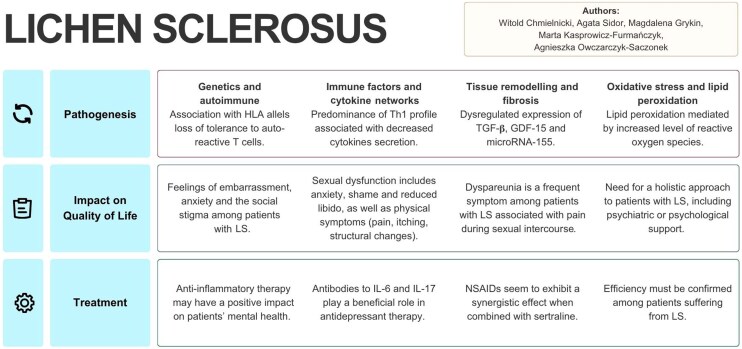

Lichen sclerosus (LS) is a chronic, inflammatory dermatological disorder that affects both sexes, with a higher prevalence in women. The anogenital region is most commonly involved,^1^ presenting as ivory white patches and plaques.^2^

LS significantly impacts patients’ physical and psychological well-being, causing pain, sexual dysfunction and reduced quality of life.^3^ Although, the ethology remains incompletely understood, immune dysregulation and persistent inflammation play a pivotal role.^1^ Overexpression of proinflammatory cytokines such as interleukin (IL)-6, tumour necrosis factor (TNF)-α and interferon (IFN)-γ may influence local tissue remodelling and systemic responses, potentially affecting neurotransmitter systems.^4^

This review explores the link between chronic inflammation, cytokine-induced neurotransmitter alterations and psychiatric disorders in patients with LS, aiming to inform more integrated therapeutic approaches.

## Materials and methods

A literature search was conducted using PubMed for publications from 2006 to 2025. Keywords included ‘lichen sclerosus’, ‘LS’, ‘inflammatory dermatosis’, ‘quality of life’, ‘DLQI’, ‘sexual function’, ‘immune factors’, ‘cytokine networks’, ‘inflammation’, ‘neurotransmission’, ‘depression’. Original research studies and review articles were considered, while conference abstracts and low-quality or inaccessible sources were excluded. Due to the narrative character of this review, the selection of publications was nonsystematic and based primarily on their substantive relevance and the currency of data.

## Pathogenesis of lichen sclerosus

### Genetics and autoimmunity

Evidence suggests that LS has an autoimmune component, as many patients present with concomitant autoimmune conditions such as autoimmune thyroiditis, vitiligo or alopecia areata.^3,5^ Additional factors supporting a genetic predisposition include family history, female predominance and higher concordance among monozygotic twins.^3^

A positive family history of LS occurs in 12% of first-degree female relatives.^6^ Particular attention has been directed towards associations with human leucocyte antigen (HLA) alleles. The *HLA-DQ7* allele has been more commonly observed in male and female patients with LS.^7^ Other HLA alleles that are important in the development of the disease include *HLA-DQ8*, *HLA-DQ9* and *HLA-DR12*, mainly the DRB1*12/DQB1*0301/04/09/010 haplotype.^8^ These alleles are thought to present self-antigens to autoreactive T cells, promoting loss of tolerance and chronic inflammation.^9^

Furthermore, microRNA-155 (miR-155) is also upregulated and promotes T-cell activation and proinflammatory cytokines ­production, while inhibiting immunosuppressive pathways by targeting molecules such as CDKN1B and FOXO3, sustaining the inflammatory cascade.^10^

### Immune factors and cytokine networks

Cellular and humoral immune responses are involved in the pathogenesis of LS with predominant T helper 1 (Th1) profile, indicated by the expression of CCR3 and CCR5 chemokine receptors by the involved cells and the absence of CCR3 and CCR4 receptors. Th1 cells secrete IFN-γ, which attracts more of these cells and intensifies the response. The secretion of other pro­inflammatory factors such as IL-1α, IL-7, IL-15, TNF-α, CD25 (IL-2 receptor), caspase-1, intercellular adhesion molecule 1 (ICAM-1) and its ligand CD11a is also increased, while the secretion of anti-inflammatory factors is reduced. The proinflammatory cytokine cascade facilitates the recruitment of inflammatory cells to the lesions by increasing the expression of chemokine receptors, including CXCR3, CXCL9-11, CCR5, CCL4 and CCL5.^7,11,12^

Recent findings highlight the involvement of the IL-17 and ­antimicrobial peptides, particularly S100A7 (psoriasin), in the pathogenesis of LS. IL-17, predominantly secreted by Th17 cells, contributes to chronic inflammation by promoting keratinocyte activation, the recruitment of neutrophils and the secretion of antimicrobial peptides such as S100A7, which further amplify local immune responses and tissue injury.^1,13^

Additionally, IL-17 synergizes with TNF-α to induce S100A7 expression in keratinocytes. Beyond its antimicrobial properties, S100A7 serves as a potent chemoattractant for leucocytes. It stimulates keratinocytes to produce additional proinflammatory cytokines, such as TNF-α and IL-8, thus creating a self-perpetuating inflammatory loop within affected tissue.^14^

### Tissue remodelling and fibrosis

Chronic inflammation induces tissue remodelling, which is characterized by the loss of elastic fibres, hyperkeratosis and dermal fibrosis in the upper dermis. This process is mediated by dysregulated expression of transforming growth factor-β and growth differentiation factor 15, accompanied by increased activity of matrix metalloproteinases (MMP-2, MMP-9) and reduced levels of ­extracellular matrix protein 1. These alterations contribute to extracellular matrix disorganization and dermal sclerosis. Furthermore, overexpression of miR-155 promotes fibroblast proliferation and collagen deposition, thereby enhancing structural rigidity and loss of tissue elasticity.^7^

### Oxidative stress

**Figure 1:**
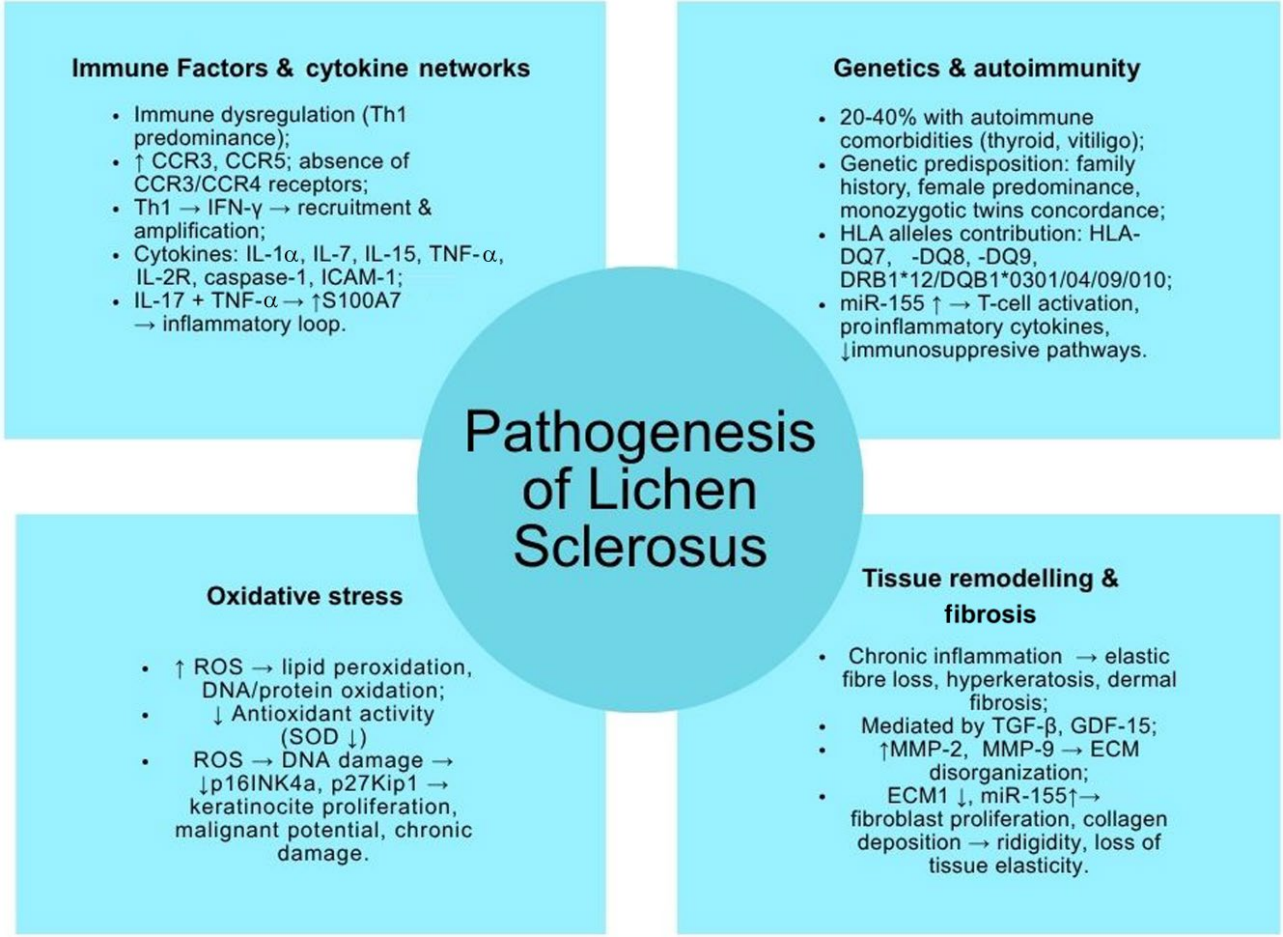
Pathogenesis of lichen sclerosus. ECM, extracellular matrix; GDF, growth/differentiation factor; HLA, human leucocyte antigen; ICAM, intercellular adhesion molecule; IFN, interferon; IL, interleukin; miR-155, microRNA-155; MMP, matrix metalloproteinase; ROS, reactive oxygen species; SOD, superoxide dismutase; Th1, T helper 1; TGF, transforming growth factor; TNF, tumour necrosis factor.

Oxidative stress is considered to play a significant role in the pathogenesis and progression of LS. Increased levels of reactive oxygen species (ROS) in skin lesion lead to lipid peroxidation, ­oxidative DNA damage and protein oxidation. These effects are compounded by a reduction in antioxidant enzyme activity, particularly superoxide dismutase. DNA damage induced by ROS downregulates the expression of two key cyclin-dependent kinase inhibitors p161^INK4^ and p27^Kip1^ thereby promoting uncontrolled keratinocyte proliferation and potentially increasing malignant potential in long-standing disease (Figure 1).^7,15^

### Urine and occlusion

Urine and occlusion are important in pathogenesis of male genital lichen sclerosus (MGLSc). Postvoiding microincontinence causes microscopic droplets of urine to accumulate between the mucosal surfaces of the glans and prepuce. Long-term urine exposure may cause lichenoid inflammation, skin thinning and sclerosis, which can cause adhesions, scarring, loss of elasticity and architectural alterations.^16,17^ Mapping studies have demonstrated that LS lesions preferentially involve areas of the prepuce and glans most exposed to residual urine, suggesting a local irritant effect related to chronic occlusion.^18^ Some researchers associate urine occlusion with expression of IL-1A, IFN-γ and IL-6 proinflammatory cytokines in the foreskin.^19^ The markedly lower incidence of MGLSc in circumcized men further supports this hypothesis, as removal of the foreskin eliminates the occlusive microenvironment and reduces chronic urine contact.^20^ Notably, complete or near-complete remission of MGLSc has been seen after urinary diversion surgeries, supporting the theory that long-term exposure to urine is a major driving factor rather than a mere consequence of the disease.^16^

## Impact of lichen sclerosus on quality of life

LS, particularly in cases involving the vulvar area (VLS), has a profound impact on daily functioning and overall quality of life in women and men. The condition frequently presents with nonspecific early symptoms, leading to delays in diagnosis and treatment, which contributes to increased patient frustration. Studies utilizing the Dermatology Life Quality Index (DLQI) have demonstrated that LS is associated with a substantial emotional ­burden.^21^ For example, a recently published Swedish study conducted a detailed comparison of DLQI scores with Lichen Sclerosus Score. A strong, statistically significant correlation was demonstrated in both sexes before treatment. Although after treatment the correlation remained statistically significant only among men, the results indicated a meaningful association between LS severity and patients’ quality of life.^22^

Patients usually report persistent and distressing symptoms such as itching, burning, stinging pain and discomfort, all of which disrupt daily activities and negatively affect mental wellbeing.^2,21^ One of the studies, focusing on women’s experiences related to LS, identified specific statements concerning sensations associated with the disease. Notably, this study was largely based on qualitative, open-ended interviews collected from female patients with LS rather than standardized psychometric tools. The patients indicated that although the disease is neither infectious nor caused by poor hygiene, due to its characteristics it is often perceived as being associated with sexually transmitted diseases. In some patients, this resulted in feelings of shame and embarrassment, as well as social withdrawal due to fear of stigmatization. An additional issue is that this condition is not something patients feel able to discuss openly with their close relations, which typically results in a lack of social support.^23^

Feelings of embarrassment and anxiety, and the social stigma surrounding discussions of genital diseases further exacerbate the psychological impact. Women tend to report more symptoms and higher perceived disease burden than men.^21^ VLS also follows a chronic course, which can lead to limited or short-term treatment efficacy and carries a risk of malignant transformation. These factors collectively contribute to the psychological burden experienced by patients.^3^ Over the long term, patients demonstrate a significantly increased risk of developing depression. In one analysis, it was demonstrated that 3.9% of patients with LS received a diagnosis of depressive episode within 5 years of LS diagnosis, compared with 3.4% of control participants during the same period. Interestingly, despite a clear deterioration in patients’ quality of life, the same study did not demonstrate an increased risk of suicidal behaviour.^24^

Further evidence from an aforementioned study involving 52 women diagnosed with VLS reported a marked reduction in quality of life expressed in DLQI, particularly in areas such as mental health, interpersonal relationships and everyday functioning. The authors emphasized that the chronic nature of LS, in combination with persistent symptoms and genital involvement, significantly impairs emotional wellbeing. The findings support the need for a holistic, patient-centred approach that includes not only medical management, but also psychological and educational support for women affected by LS.^25^

## Impact of lichen sclerosus on sexual function

Studies suggest that more than half of women and men with LS report experiencing pain during sexual intercourse, leading to dyspareunia or, in more severe cases, complete avoidance of sexual activity (apareunia). Among women, this pain is often caused by erosions, fissures and anatomical changes due to chronic inflammation.^21^ These changes are reflected in the results of standardized tests assessing patients’ sexual life. In one Polish study, The Sexual Quality of Life–Female (SQOL-F) administrated to female patients demonstrated a significant reduction in sexual quality of life compared with the control group. This may result from the aforementioned physical pain caused by dermatological lesions. However, an important contributing factor is also the emotional discomfort associated with negative self-perception and diminished sense of attractiveness.^26^ These changes not only compromise sexual activity, but also negatively affect body image. This may concern not only current, but also potential sexual partners. Patients’ statements indicate that new acquaintances frequently perceive the disease as infectious, which leads them to refrain from initiating sexual relationships. Furthermore, the appearance of the lesions and the associated feelings of shame prevent patients from engaging in sexual relationships with new partners.^23^

Men frequently experience notable improvement after circumcision, which can alleviate physical symptoms and enhance sexual comfort. However, in women, particularly those with severe disease, psychosexual difficulties may persist, despite appropriate treatment.^21^ Nevertheless, it should be borne in mind that psychosexual counselling has been shown to play a significant role in the treatment of female patients with LS. Through sexological therapy, women gained greater self-­confidence in engaging in sexual intercourse. It has been also demonstrated that the involvement of the partners of patients with LS in therapy may positively contribute to improving their mutual sexual relationship.^25^ Sexual dysfunction encompasses physical symptoms (pain, itching, structural changes) and psychological components (anxiety, shame, reduced libido).

In the cited study, researchers also identified sexual health as one of the most affected domains in women with VLS based on DLQI scores. Participants frequently reported reduced sexual satisfaction and avoidance of sexual activity due to fear of pain, embarrassment and low self-esteem. The authors concluded that psychological counselling and sexual education, when offered alongside clinical care, can improve coping strategies and contribute to better sexual functioning and quality of life.^25^

## Sex-specific presentation

Apparent anatomical differences between the sexes may contribute to sex-specific clinical presentations and complications with LS. Particular consideration should be given to men with concomitant phimosis, in whom the diagnostic process may be further complicated, as clinical focus is often directed primarily towards the surgical management of phimosis. Early manifestations of LS may be subtle, and concentrating solely on phimosis-­related symptoms may delay diagnosis. Moreover, in such cases, LS-related changes involving the glans penis may be obscured by the foreskin. Failure to recognize these features may subsequently complicate treatment and increase the risk of adverse outcomes, including meatal sclerosis or squamous cell carcinoma.^27^

In men with concomitant phimosis and those without comorbid conditions, LS is associated with impairments in quality of life, including sexual wellbeing. For example, in an Italian study evaluating the sexual quality of life among men with LS, those with pemphigus and healthy control participants demonstrated significantly lower sexual satisfaction in the LS group, using the International Index of Erectile Function. Deficits were observed across several domains, including erectile function, orgasm, sexual desire and overall sexual satisfaction. Statistical analysis indicated that these impairments were primarily related to disease-associated pain.^28^ Nonetheless, additional symptoms such as pruritus, dysuria and fissures may also clinically contribute to diminished sexual wellbeing, despite the lack of statistical significance reported in that study.

In this study, women were likewise included in the analysis. Despite experiencing difficulties with orgasm, sexual desire and overall satisfaction similar to men, the female-specific Female Sexual Function Index additionally revealed impairments in arousal and lubrication that were not identified in the male cohort. These outcomes were likewise inferior compared with those observed in patients with pemphigus and in the healthy control group. In this case, statistically significant associations were identified between sexual dysfunction and symptoms such as pruritus, pain, dysuria and whitening. Anatomical changes, including clitoral hood fusion, as well as labial fusion or resorption, were also found to be significant.^28^

## Correlation between lichen sclerosus and inflammation

The overproduction of proinflammatory cytokines and mediators such as IL-1, IL-7, IL-15, IFN-γ, TNF-α, CD25 (IL-2 receptor), caspase-1, ICAM-1 and its ligand CD11a, plays a particularly important role in the pathophysiology of LS.^1^ Some of them, such as IL-1 (IL-1β), IFN-γ and TNF-α, are described as factors that are involved in the inflammatory theory of depression, which could suggest a potential explanation for the increased risk of depressive disorders and decreased quality of life among patients with LS.

### The role of inflammation in depression

Depression is a psychiatric disorder affecting an estimation of 300 million people from all age categories.^29^ It is characterized by psychological, behavioural and physiological symptoms that include irritability, pessimism, mood reactivity, insomnia, weight loss and loss of sexual interest.^30^ Currently, numerous theories explaining the development are either well-established or under consideration. These include the monoamine hypothesis, the hypothesis connected with decreased melatonin level, the neuro­plasticity dysregulation hypothesis and the inflammation hypothesis in depression.^31^ In the context of LS, as an inflammatory disease, the last of the aforementioned theories is of particular significance.

In the course of LS, overactivation of the inflammatory system is observed. The infiltrate of activated T cells releasing IL-4 and overproduction of IL-1 constitute a basis of the disease.^32^ Increased production of IL-12, IL-2, IL-5 and IL-10 by lymphocytes of patients with LS has also been observed.^33^ It seems that these cytokines can modulate the synthesis and release of neurotransmitters involved in mood regulation.^31^

In this context, the accessibility of neurotransmitters is closely linked to the kynurenine pathway (KP). L-Tryptophan is a precursor of serotonin, a monoamine neurotransmitter that modulates the activity of nervous system.^34^ Under the action of indoleamine 2,3-dioxygenase (IDO) 1 and 2 tryptophan is converted into *N*-formyl-L-kynurenine and subsequently transformed into L-­kynurenine. The activity of IDO is known to be intensified by proinflammatory cytokines such as TNF-α, IFN-γ, IL-1 and IL-6. Moreover, it has been proven that the synergistic effect between IFN-γ and IL-1 affects IDO enzyme activity and transcription. It should be emphasized that numerous studies investigating the effects of individual cytokines on KP activity have been conducted on animal models.^35^ Nevertheless, when considered alongside findings from studies conducted in humans, they provide arguments supporting the hypothesis that increased activity of those cytokines may lead to a diminished level of serotonin and dysregulation of the serotonergic system. Moreover, the products of tryptophan degradation are also considered as factors causing neurotoxic effect.^31^

### Role of tetrahydrobiopterin and reactive oxygen species in neurotransmitter synthesis

The literature indicates a significant role of tetrahydrobiopterin (BH4), which is the essential cofactor in the synthesis of serotonin, dopamine and other catecholamines.^36^ BH4 is also a cofactor for nitric oxide synthase (NOS). The level of NOS increases significantly during inflammation, which may contribute to oxidative stress and formation of ROS. In the presence of ROS, BH4 is reduced to dihydrobiopterin (BH2) and the BH4 availability may be limited, leading to a reduction of dopamine synthesis. The association between reduced BH4 levels and inflammation has been confirmed in studies conducted in both animals and humans.^37^

ROS play a significant role in numerous inflammatory diseases, including LS. Oxidative stress has an impact not only on pathogenesis, but also on the development, maintenance and progression of LS. Excess ROS may cause oxidative DNA damage, oxidative protein damage or concentration of lipid peroxidation products in keratinocytes of the epidermal basal cell layers.^15^ Moreover, oxidative stress is exacerbated by proinflammatory cytokines,^37^ including those for which an increased level is observed among patients with LS. As described above, this may affect neurotransmission. However, the literature lacks information on whether exactly the same mechanism occurs in such group.

## Impact of peripheral inflammation on neurotransmission

Dysregulation in serotonergic neurotransmission may cause poor mood, changes in appetite, changes in sleep, and sexual and cognitive dysfunction. Anhedonia, reduced psychomotor speed, impaired concentration and lack of motivation may be caused by dopamine impairment. However, dysregulation of the noradrenaline system is believed to result in low energy, attention and concentration deficits, and reduced cognitive ability.^37^

Described inflammation-related mechanisms may be associated with monoamine signalling. Proinflammatory cytokines trigger dysregulation of monoamine synthesis through the described mechanisms. Monoamine reuptake may also be disrupted by the impact of proinflammatory cytokines (Il-1β, TNF-α and IFN-γ) on monoamine transporters (MATs). For instance, such cytokines upregulate serotonin transporter activity, leading to a decreased level of serotonin. MATs are also regulated by numerous signalling pathways that are activated by IL-1β or TNF-α. Reuptake of serotonin, dopamine or noradrenaline could be significantly stimulated. Inflammation also affects the release and metabolism of neurotransmitters. For example, it has been experimentally demonstrated that IL-1β increases the release of dopamine, noradrenaline and serotonin, including their metabolites. However, this association appears to have been demonstrated primarily in murine models.^4^ All of those processes seem to result in impaired neurotransmission, and consequently, in the development of mental disorders. It is worth emphasizing that, despite studies conducted in recent years in this area, the results remain indirect. Nevertheless, the association between immunological factors and disturbances in neurotransmission offers considerable promise for a broader perspective on mental disorders in patients with LS.

## Treatment

The association between inflammation and depressive disorders raises the question of whether anti-inflammatory therapy may lead to improvement in affected patients. Several studies have suggested that anticytokine medications may have an impact not only on inflammation, but also on mental condition. A particular role is attributed to anti-TNF-α treatment. Although medications such as infliximab do not demonstrate efficacy comparable to antidepressants, they may lead to improvement among patients with high baseline inflammatory biomarkers. Nevertheless, one study showed that by using etanercept, adalimumab and golimumab among patients with psoriasis, the rate of antidepressants use may be reduced.^38^

Although antibodies to TNF-α are the most studied anticytokine therapeutic in depression, antibodies to IL-6 and IL-17 show some promise as well. It has been demonstrated that patients with psoriasis treated with sirukumab and situximab (antibodies to IL-6), and ixekizumab (antibody to IL-17) showed improvement in depressive symptoms. The result was compared with diminished C-reactive protein value.^39^ Additionally, the impact of ixekizumab treatment on depressive symptoms and systemic inflammation was thoroughly examined in patients with moderate-to-severe psoriasis. Compared with placebo, patients who received ixekizumab demonstrated significant improvement of mental health. The treatment had a positive impact on sad mood, self-criticism, energy/fatigue and retardation. A potentially beneficial effect of ixekizumab on reducing the frequency of suicidal thoughts and attempts in this group has also been indicated. However, the efficacy of nonsteroidal anti-inflammatory drugs is also considered. For example, celecoxib, a cyclooxygenase-2 inhibitor, seems to exhibit a synergistic effect when combined with sertraline. Patients treated with both medications exhibit a faster therapeutic response compared with the placebo group that did not receive celecoxib.^40^ Nevertheless, it should be emphasized that the results obtained concerned patients with psoriasis. Currently, there is a lack of data on whether such therapies would be effective in patients with LS. These results should therefore be regarded as potential directions for research in this area.

## Data Availability

The data underlying this article will be shared on reasonable request to the corresponding author.

## Conclusions

LS presents a challenge not only from a dermatological perspective, but also from psychiatric and psychological standpoints. The complexity of the pathogenesis suggests a possible association between inflammatory processes and psychiatric disorders. Particular attention is drawn to proinflammatory cytokines such as IL-1 (IL-1β), IFN-γ or TNF-α and their impact on the metabolism of neurotransmitters. However, there are insufficient data to establish a definitive correlation. Nevertheless, studies dedicated to the cytokine theory of depression and the impact of anti-inflammatory treatment on mental health offer hope for identifying potential links related to LS. Confirmation of such a hypothesis would have a positive impact on patients in this group by improving their quality of life and reducing the psychiatric disorders associated with LS.
